# Patients' views and experiences of live supervised tele‐exercise classes following bariatric surgery during the COVID‐19 pandemic: The BARI‐LIFESTYLE qualitative study

**DOI:** 10.1111/cob.12499

**Published:** 2021-11-28

**Authors:** Friedrich C. Jassil, Rebecca Richards, Alisia Carnemolla, Neville Lewis, Gemma Montagut‐Pino, Helen Kingett, Jacqueline Doyle, Amy Kirk, Adrian Brown, Kusuma Chaiyasoot, Kalpana Devalia, Chetan Parmar, Rachel L. Batterham

**Affiliations:** ^1^ Centre for Obesity Research University College London London UK; ^2^ Bariatric Centre for Weight Management and Metabolic Surgery University College London Hospitals NHS Trust London UK; ^3^ National Institute for Health Research UCLH Biomedical Research Centre London UK; ^4^ MRC Epidemiology Unit University of Cambridge Cambridge UK; ^5^ The Hatter Cardiovascular Institute, Institute of Cardiovascular Science University College London London UK; ^6^ Bariatric Surgery Department Homerton University Hospital NHS Trust London UK; ^7^ Department of Surgery Whittington Health NHS Trust London UK

**Keywords:** bariatric surgery, COVID‐19, exercise, physical activity, qualitative, telehealth

## Abstract

The BARI‐LIFESTYLE trial is a randomized controlled trial evaluating the efficacy of a post‐surgery nutritional and behavioural tele‐counselling, and supervised exercise programme to maximize the health benefits of bariatric surgery. Due to the coronavirus disease 2019 (COVID‐19) pandemic, the in‐person supervised exercise component had to be converted to remote tele‐exercise. However, patients' acceptability of this method of exercise provision is unknown. Between 3 and 6 months following bariatric surgery, 13 adults participated in weekly, structured, 60‐min supervised exercise classes delivered via Zoom by a trained exercise therapist. A total of 12 participants (*n* = 8 female), with a mean age of 46.3 (range 33–63) years, who had undergone either sleeve gastrectomy (*n* = 8) or Roux‐en‐Y gastric bypass (*n* = 4) surgery, participated in one‐to‐one semi‐structured interviews following the tele‐exercise classes. Interviews were audio‐recorded, transcribed verbatim and analysed using thematic analysis. Participants described how the tele‐exercise classes helped them to cope with the changes to their lives brought about by the COVID‐19 pandemic. Participants found the tele‐exercise schedule, content and intensity to be acceptable, and were satisfied with the privacy, security and safety of the technology and classes. Professional supervision and guidance from an exercise therapist were described as central to the tele‐exercise provision. Importantly, participation in the tele‐exercise provided physical, emotional and social benefits. Few participants reported barriers to participation. Overall, the tele‐exercise classes were deemed acceptable and compared favourably to in‐person exercise classes.

**What is already known about this subject**

- Exercise provides additional health benefits after bariatric surgery; however, most patients do not meet the recommended level of physical activity.
- Post‐surgery supervised exercise programmes have been shown to maximize the health outcomes of bariatric surgery, but low uptake is common due to barriers such as geographical accessibility of exercise facilities and lack of time.
- The imposed restrictions along with limited access to bariatric care service during the COVID‐19 pandemic have led to an increased level of anxiety, decreased level of physical activity and weight gain.
- Tele‐exercise can increase accessibility to structured exercise programmes; however to date, no study has explored in‐depth views and experiences of patients who have undergone bariatric surgery towards a remotely delivered supervised exercise programmes.

**What this study adds**

- Post‐bariatric tele‐exercise classes were feasible and acceptable to patients and compared favourably to in‐person exercise classes.
- The tele‐exercise classes helped patients to cope with the extra challenges associated with the COVID‐19 pandemic in adhering to the lifestyle changes recommended following bariatric surgery.
- Useful suggestions were identified to optimize the delivery and safety of, and adherence to, tele‐exercise classes, which could be considered when planning and developing future tele‐exercise programmes for patients pre‐ and post‐bariatric surgery.

## 1 INTRODUCTION

Bariatric surgery is currently the most effective treatment option for people living with severe obesity^1^; however, approximately 20%–33% of patients experience poor weight loss outcomes and weight regain over the long term.^2^, ^3^, ^4^ Exercise provides additional health benefits after bariatric surgery^5^ but the majority of patients do not meet the recommended level of physical activity and studies suggest that more than 60% of their waking time is spent in sedentary behaviour.^6^, ^7^, ^8^, ^9^, ^10^ Post‐bariatric exercise services help patients to adopt and/or maintain exercise behaviours however, access to these is limited.^11^ In the United Kingdom, most bariatric centres do not offer exercise programmes as part of standard post‐surgery care due to the lack of evidence‐based research to support their implementation.^12^ Therefore, we conducted the BARI‐LIFESTYLE randomized controlled trial (RCT) to evaluate the efficacy of a combined nutritional–behavioural tele‐counselling and supervised exercise programme in the first year after bariatric surgery (ClinicalTrials.gov Identifier: NCT03214471).

In March 2020, the UK government imposed a nationwide lockdown to contain the spread of the coronavirus disease 2019 (COVID‐19),^13^ which we now know had a negative impact on mental health and health‐related behaviours such as physical activity, especially for people living with obesity.^14^ All research‐related in‐person activities were suspended to abide with the restrictions. In order to maintain the integrity of BARI‐LIFESTYLE, the supervised exercise component was modified to be delivered remotely via Zoom, a cloud‐based video conferencing service (referred to hereafter as tele‐exercise).^15^ With the advancement in digital communication technology, health systems worldwide are looking to integrate online delivery of services to improve the overall efficiency and effectiveness of care.^16^ The acceptability of such technology for patients is essential for the successful implementation and uptake of such services. To date, the use of telehealth to deliver exercise programmes in patients pre‐ and post‐bariatric surgery is scarce.^17^ In view of this, the present study sought to: (1) explore experiences and views of patients who have undergone bariatric surgery on supervised tele‐exercise classes, (2) identify the barriers to, and limitations of such classes, and (3) identify points of intervention that could be targeted to optimize the delivery and safety of, and adherence to, a future tele‐exercise programme.

## 2 MATERIALS AND METHODS

### 2.1 Study design and participants

This qualitative study is an additional sub‐study of the BARI‐LIFESTYLE trial that received ethical approval by London‐Dulwich Research Ethics Committee (17/LO/0950) as part of a protocol amendment. The use of semi‐structured interviews as exploratory method provided a wealth of raw data that is particularly useful in assessing needs and informing the design for future interventions. Semi‐structured interviews were selected to ensure that specific research questions were addressed; however, participants were retained the freedom to bring up other topics if they felt they were important to the study. Data were analysed using thematic analysis, which involves identifying and making sense of patterns that emerge from qualitative data by organizing them into meaningful themes.^18^ Because thematic analysis adopts an inductive approach, it is particularly useful when studying under‐researched areas where there is insufficient knowledge to apply meaningful theories or hypotheses a priori, which is the case in this present study of bariatric surgery patients' perspectives on tele‐exercise. This study was carried out by the Centre for Obesity Research, University College London (UCL).

**FIGURE 1:**
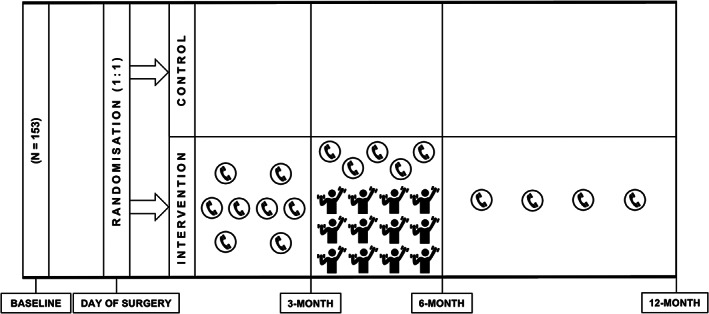
Schematic representation of BARI‐LIFESTYLE trial. The lifestyle intervention programme consisted of a nutritional‐behavioural tele‐counselling with dietitian and once weekly supervised exercise classes in the hospital gym for 12 weeks

In brief, BARI‐LIFESTYE was a multi‐site, two‐arm, parallel group, single‐blinded RCT embedded within an observational cohort. Participants were recruited from University College London Hospitals (UCLH), Whittington Hospital and Homerton University Hospital. A total of 153 participants were enrolled in the BARI‐LIFESTYLE observational study since March 2018.^19^ On the day of surgery, all participants in this cohort were randomized (1:1 allocation) to either receive post‐surgery standard care or standard care plus a lifestyle intervention (BARI‐LIFESTYLE intervention study) (Figure 1).

**FIGURE 2:**
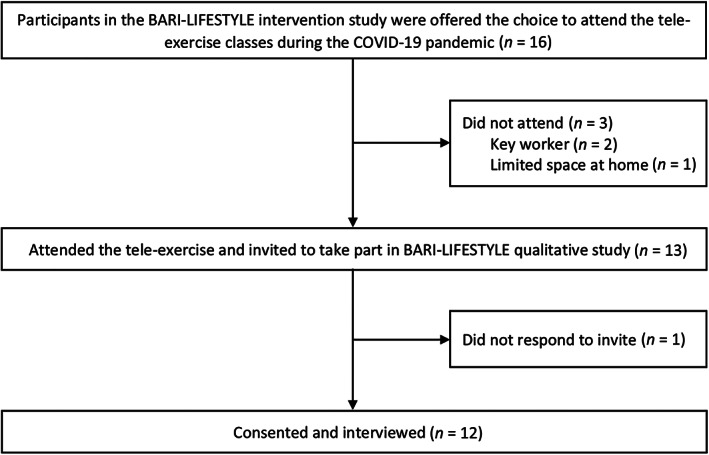
Participant flow diagram

By the time the UK government announced the stay‐at‐home order,^13^ a total of 16 remaining participants in the intervention group were still actively participating in the gym exercise class. Of these, 13 participants agreed to complete the remaining exercise sessions remotely via Zoom (Zoom Video Communications, Inc., California, USA). Two months after the end of the tele‐exercise classes, these 13 participants were invited to take part in qualitative interviews via phone call and/or email, or an invitation letter to those who could not be reached. Interested subjects were given a copy of the participants' information sheet for the qualitative study, detailing what the study entails, and encouraged to contact the research team should they have further questions related to the study. To be eligible for inclusion, participants must have had attended at least three tele‐exercise classes to ensure they could provide in‐depth insights into the tele‐exercise programme. Of all eligible participants approached, 12 participants agreed to be interviewed (Figure 2).

### 2.2 Tele‐exercise classes

Participants were invited to attend one of the three weekly tele‐exercise classes, delivered via Zoom in a group format (Tuesday at 10:30 AM, Thursday at 5:30 PM and Saturday at 10:30 AM). To assist the organization of the classes, a group messaging app, WhatsApp (Facebook, Inc., California, USA) was set up. The tele‐exercise class content (Supporting Information S1) was designed based on our experiences in a previous pilot feasibility study.^20^ A total of 45 classes were carried out throughout the lockdown period from April to July 2020. The overall attendance in each class ranged between two to six participants.

### 2.3 Topic guide

The interview topic guide was developed using the research questions and review of previous qualitative literature that explored the use of telehealth to deliver exercise programmes^21^, ^22^ and exercise interventions for patients who have undergone bariatric surgery.^23^, ^24^ Questions focused on exploring participants' overall experiences and views of the tele‐exercise classes, including the use of technology, the content of the classes, the exercise therapist and supervision; identifying barriers and facilitators of participation, benefits and/or limitations of the classes; and identifying elements for future improvements of the classes (Supporting Information S2). The interview questions were tested and revised in the first three interviews to ensure participants were able to comprehend the questions. These three interviews were included in the analysis.

### 2.4 Data collection

The lead author (FCJ) recruited and conducted 12 individual, in‐depth, semi‐structured interviews with participants, of which seven were conducted in‐person, three by telephone, and two by video call using Zoom. All in‐person interviews took place at UCLH. Written informed consent for participation in the study was obtained prior to the face‐to‐face interviews. Whereas for the telephone and video call interviews, the consent forms were either: (1) posted to the participants' home address and the signed consent forms were returned using a stamped addressed envelope provided or (2) emailed to the participants and the signed consent forms were emailed back, prior to the interviews undertaken. All interviews were audio‐recorded and anonymized using the same unique PIN number assigned in the initial RCT, participants consented to the audio‐recording when signing the consent form. Interviews were conducted between October and December 2020 with interview lasting between 23 and 46 min (mean of 33 min). All interviews were transcribed verbatim and checked against the recordings for accuracy.

### 2.5 Data analysis

Transcripts were analysed using an inductive form of thematic analysis^18^ using NVivo 12 (QSR International Pty Ltd., version 12, 2014) to provide a detailed and data‐driven account of participant's view and experiences. Given the limited knowledge of patients' views and experiences of live supervised tele‐exercise classes following bariatric surgery, the aim of the current study was not to test a specific theory, but rather to take an inductive approach that identified points of particular salience in patients' own accounts of their experience. Reflexivity was maintained by keeping a research journal and by regular discussion among the researchers (FCJ and RR) to help manage pre‐assumptions and cross‐check that the analysis was reflective of the data. Initially, two researchers (FCJ and RR) independently read four transcripts to familiarize themselves with the data and coded the transcripts line‐by‐line. Both FCJ and RR met weekly to discuss their preliminary codes and refined them through an iterative process until a consensus was reached. Using this initial framework of codes, FCJ then continued coding the remaining transcripts. FCJ and RR continued to meet weekly to discuss new codes and refine them, until no more new codes were generated from the data. Next, FCJ and RR independently extracted the codes that shared similar ideas and concepts to represent broader level categories that held relevance to the research questions. Both FCJ and RR met to discuss their framework of categories and refined them through an iterative process until a consensus was reached. The reviewed categories were organized into potential themes or sub‐themes. Next, the codes and themes were reviewed and refined to ensure that the themes demonstrated a valid, accurate and coherent pattern. When all themes were finalized, the names of the themes were refined to check that they provided a valid account of the data that they represent. Specific quotations were extracted to illustrate the themes and subthemes. FCJ is a dietitian and involved in BARI‐LIFESTYLE trial. RR is a health psychologist with training and experience in conducting and analysing qualitative interviews. RR was not involved in the wider BARI‐LIFESTYLE trial.

## 3 RESULTS

**FIGURE 3:**
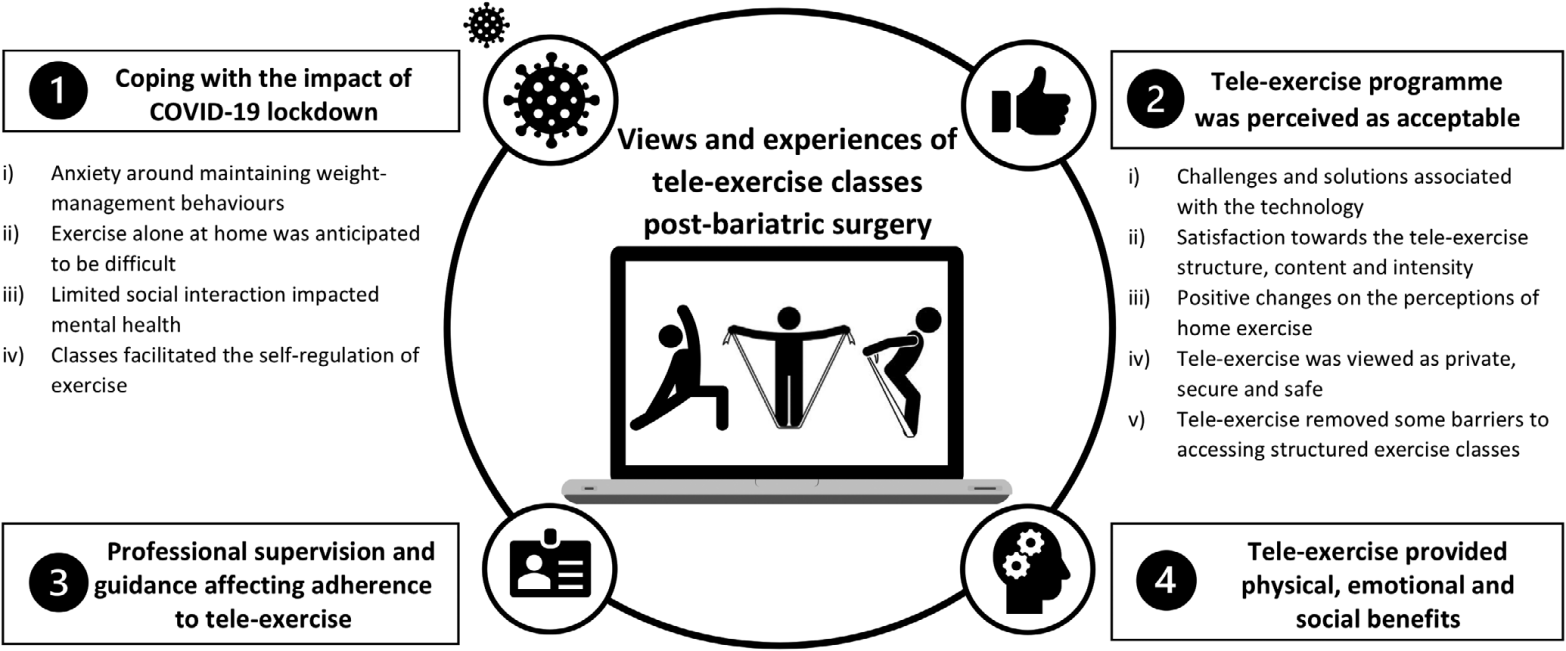
Visual representation of themes and sub‐themes

Participants' characteristics are presented in Table 1. Four overarching themes were generated from the data (Figure 3). Additional examples of quotations for themes and subthemes are also presented in Supporting Information S3.

**TABLE 1:** Summary of participants' characteristics

Participant characteristics	Total samples, *n* = 12
Gender, *n* (%)	
Male	4 (33.3)
Female	8 (66.7)
Age (years), *n* (%)	
30–40	5 (41.7)
41–50	2 (16.7)
51–60	4 (33.3)
61–64	1 (8.3)
Mean (years)	46.3
Range (years)	(33–63)
Ethnicity, *n* (%)	
Asian or Asian British	3 (25)
Black or Black British	2 (16.7)
White or White British	7 (58.3)
Education level, *n* (%)	
No qualification	1 (8.3)
A level or equivalent	1 (8.3)
University degree	6 (50)
Higher degree	4 (33.3)
Employment status, *n* (%)	
Employed	9 (75)
Unemployed	1 (8.3)
Others	2 (16.7)
Study site, *n* (%)	
UCLH	6 (50)
Whittington	3 (25)
Homerton	3 (25)
Type of surgery, *n* (%)	
RYGB	4 (33.3)
SG	8 (66.7)
Exercise classes attended, *n* (%)	
Gym exercise (face‐to‐face)	
<50%	8 (66.7)
≥50%	4 (33.3)
Mean (day)	5
Range (day)	1–8
Tele‐exercise (virtual)	
<50%	5 (41.7)
≥50%	7 (58.3)
Mean (day)	7
Range (day)	3–11

Abbreviations: *n*, number; RYGB, Roux‐en‐Y gastric bypass; SG, sleeve gastrectomy; UCLH, University College London Hospitals.

### 3.1 Theme 1: Coping with the impact of COVID‐19 lockdown

Most participants described how the COVID‐19 pandemic had negatively impacted many aspects of their daily routines, which made it challenging to adhere to the post‐bariatric surgery lifestyle recommendations. Being enrolled in the tele‐exercise classes was perceived to have helped them to cope with the changes brought about by lockdown.

#### 3.1.1 Sub‐theme (i): Anxiety around maintaining weight‐management behaviours

Due to the restrictions imposed during lockdown, such as gym closures and changes in routines, some participants felt anxious about being able to maintain their weight‐management behaviours, such as healthy eating and exercise, in line with their post‐surgery recommendations. This led to a fear of weight regain.

“I thought when the gyms closed, that's it. I can't exercise, my diet is ruined. I'm going to gain a lot of weight.” (P3, Male, 52)

#### 3.1.2 Sub‐theme (ii): Exercise alone at home was anticipated to be difficult

In addition, a few participants anticipated that having to exercise at home, alone, would have been difficult due to having no one to properly guide or monitor them. Furthermore, the lockdown appeared to negatively impact their motivation to stay physically active. As a result, these participants felt that having access to the tele‐exercise classes provided them with the much‐needed support to engage in physical activity and helped to increase their motivation to keep active during this challenging period.

“Having the class online really felt very supportive, motivating and very helpful in order to keep me on track exercising. I had the support because to do it on my own, without someone in real‐time being there, I find more difficult.” (P5, Male, 63)

#### 3.1.3 Sub‐theme (iii): Limited social interaction impacted mental health

Many participants described how the limited face‐to‐face social interaction during lockdown had also negatively impacted their mental health. The tele‐exercise classes gave them something to look forward to and enabled them to interact with others, as well as expand their social networks, which appeared to help them cope with the social isolation caused by the pandemic.

“It helped with mental health when in lockdown. You had something to look forward to on a Saturday and you talk to other people and meet other people even though you weren't actually going out anywhere.” (P6, Female, 48)

#### 3.1.4 Sub‐theme (iv): Classes facilitated the self‐regulation of exercise

Several participants reported a loss of sense of control due to the many changes caused by the pandemic and the associated uncertainty. For example, they struggled to maintain their regular exercise routines. The tele‐exercise classes appeared to facilitate the self‐regulation of exercise by implementing a temporal and physical structure within participants' daily routines, as well as providing a source of accountability and motivation. Some participants described that having the tele‐exercise classes booked in their schedule had held them accountable to their exercise routine and promoted commitment, which may otherwise be difficult when exercising on their own.

“I find it very helpful to have it as a reference point in my diary rather than to say, okay tomorrow I will exercise, and then tomorrow finishes and I didn't exercise.” (P5, Male, 63)

Furthermore, the group‐format of the classes created further accountability that was perceived to be an important factor that would help them to achieve better results over long term. Being part of a small group, as well as knowing the therapist was waiting in class, encouraged them to turn up to sessions for fear of embarrassment that their absence would have been noticed.

### 3.2 Theme 2: Tele‐exercise programme was perceived as acceptable

All participants appreciated the invitation to tele‐exercise classes and the majority reported having a positive experience. Participants found the tele‐exercise schedule, content and intensity to be acceptable, and were satisfied with the privacy, security and safety of the technology and classes.

#### 3.2.1 Sub‐theme (i): Challenges and solutions associated with the technology

None of the participants reported experiencing any major technical difficulties in setting up the Zoom software as the majority of participants had used Zoom during lockdown. Nevertheless, several minor issues were reported by a few participants, such as the difficulty to remember the ID and password hence causing them to enter the classes late and missed the earlier part of the exercise. Furthermore, a few participants mentioned the inconveniences when accessing the classes using a phone due to the small screen size. Some participants also experienced a problem with poor internet connection that led to audio and video lagging. This happened either due to having a low internet bandwidth or when trying to access the classes outdoors (e.g., the yard, balcony).

“When my internet speed gets slower, there was a bit of lagging, but that again improved because all I needed is to get my internet upgraded. It's inevitable. There will be some tuning issues when you first start using technology for things like this.” (P4, Female, 42)

Several practical solutions were suggested by a few participants to ensure a smooth conduct of the classes, such as having a device with larger screen (e.g., desktop, laptop or tablet), a minimum internet bandwidth and instruction for them to follow.

#### 3.2.2 Sub‐theme (ii): Satisfaction towards the tele‐exercise structure, content and intensity

Almost all participants described how they particularly liked the structure of the tele‐exercise classes, for example, how they included a warm‐up and cool‐down, and a variety of cardiovascular and resistance exercises at different levels of intensity. In addition, participants perceived the exercises to be challenging but enjoyable and felt they did more exercise in classes than they would have done alone at home.

“Most of the sessions really delivered within my expectation and were very pleasant but still work. There was the warmup and then the work which involved working on muscle strength with the bands targeting different parts of the body and also cardio. And then the ending which was stretching and relaxing. So, all in all, it was well structured.” (P5, Male, 63)

Physical fitness and ability varied between participants. As a result, a small number of participants felt that some of the exercise routines were not challenging enough. In contrast, one participant reported the exercises were too challenging as he did not get enough rest time in between the routines.

“I think because you are doing it without a break, that's another issue because you are not 100% fit. Try to fit in so many activities within 60 minutes without a break, it's a bit too much.” (P2, Male, 58)

Based on the intensity of the tele‐exercise classes and that participants felt fatigued at the end of classes, the length of the classes was considered to be *“just right” (P8, Female, 55)*. Nevertheless, a minority of participants felt that slightly shorter classes would have been sufficient, while one participant wanted longer than an hour due to increased energy at the end of the class.

When participants were specifically asked about their preferences between the tele‐exercise and in‐person exercise classes, the responses were mixed with almost half of them still preferring the in‐person gym classes. This was mainly because they enjoyed working out in a gym environment, felt they had better supervision in terms of having someone watching over their techniques when using the gym equipment and valued the in‐person social interaction with peers. It is also useful for people who have not used the gym as it helped them to be confident when exercising in the gym. Nevertheless, most participants believed the tele‐exercise classes were comparable to in‐person classes in terms of keeping them on track with physical activity and perceived that they would therefore be useful for patients who are unable to attend in‐person classes. As a result, a few participants have suggested including both in‐person and tele‐exercise classes in future programmes as it may increase attendance.

“I think having a mix of both is a more effective way for the programme. It would definitely help when people couldn't attend a face‐to‐face session. That might also improve how much time people attend.” (P4, Female, 42)

#### 3.2.3 Sub‐theme (iii): Positive changes on the perceptions of home exercise

Due to having no previous experience of home workouts delivered via technology, a few participants appeared to have preconceived expectations that home‐based workouts would not be as effective as in‐person workouts in a gym. However, after attending the tele‐exercise classes, these participants were positively surprised by the level of exertion they experienced which they perceived to be as invigorating as the in‐person exercises classes. Participation in the tele‐exercise classes taught them that they do not need gym equipment, nor a large space at home, to have an effective workout. As a result, one participant commented how tele‐exercise could be useful not just for lockdown but could be integrated into the post‐surgery programme.

“I think it opened up the possibility that is something that does not necessarily only apply when you have a lockdown or social distancing. But it could be integrated in the programme at almost no additional cost to exercising in the gym.” (P5, Male, 63)

#### 3.2.4 Sub‐theme (iv): Tele‐exercise was viewed as private, secure and safe

One of the advantages of tele‐exercise classes that was reported by a few participants was feeling less self‐conscious and intimidated by their peers, compared to attending in‐person classes at the gym. These participants did not feel they were being judged because of their physical limitations or feel they were in competition with others in the tele‐exercise classes.

“When I was big, I lack a lot of confidence. So, I didn't like people to watch me do things. When I was doing it at home, I felt comfortable. Definitely more confident at home in your own environment. I think more people would want to do it that way.” (P6, Female, 48)

Although participants appeared aware of the privacy and security risks associated with using any type of virtual platform, they generally felt that the tele‐exercise classes did not require any additional security above and beyond other activities that they have had previously performed on a virtual platform. Most participants were also familiar with Zoom software, which they believed to be fairly secure. However, two participants suggested that the use of a hospital‐based virtual platform would reassure participants that their privacy is being well protected.

Participants also generally perceived the tele‐exercise classes to be physically safe, as they did not feel pressured to do exercises that they deemed as unsuitable for themselves. Additionally, participants appreciated having the instructor therapist guiding their technique and providing alternative exercises when needed, in order to prevent injuries.

#### 3.2.5 Sub‐theme (v): Tele‐exercise removed some barriers to accessing structured exercise classes

The tele‐exercise classes appeared to increase the accessibility of exercise by removing some of the barriers that are commonly associated when attending in‐person gym classes, such as geographical accessibility of exercise facilities, travel time, parking issues and poor weather conditions. Furthermore, participants appreciated the range of tele‐exercise classes offered, such as morning and evening options and weekdays and weekends, which also facilitated attendance. For example, two participants were able to join the tele‐exercise from abroad. Several participants, however, suggested that the evening sessions could be moved to a later time to allow them to attend the classes after work.

“I have a long way to travel, it's two buses to get there and if the weather is bad, you really don't want to go out. But if you're doing it online, it doesn't matter what the weather is like, you can just get on and do it.” (P6, Female, 48)

Nevertheless, some barriers to attending the tele‐exercise classes were reported by some participants, including busy work schedules, caring for family, competing priorities and illness or injury. In addition, a few participants reported that poor mental health had also led to reduced motivation for attendance on occasion.

“I injured my shoulder, and my daughter was sick, so not any other reasons behind that. I would love to attend but sometimes not everything is under your control, especially health wise and family related things.” (P10, Female, 35)

As a result, the majority of participants valued the recorded tele‐exercise session as it enabled them to attend the class even if they had missed the live session. Additionally, they could repeat the class to remind themselves of the exercises, if needed. Even though there are existing exercise videos freely available online, some participants explained that the recording of the tele‐exercise class was particularly valuable as it was specifically tailored to their needs as a post‐bariatric surgery patient.

“You can redo it [from the recordings] when you want to or when you have time. You don't remember all the time, what exercise you've done. Because even if I want to repeat what I've done on Saturday, I don't remember all of them. That is the advantage of the tele‐exercise.” (P1, Female, 39)

### 3.3 Theme 3: Professional supervision and guidance affecting adherence to tele‐exercise

All participants valued the professional supervision and guidance from an exercise therapist, which appeared to increase participants' motivation to exercise and attend the tele‐exercise classes. Most of the participants told of the challenges of being physically active following surgery, such as having poor exercise knowledge, fear of injury and poor motivation and confidence to exercise. Receiving prescriptive exercise from a professional, who was able to supervise and guide them, was therefore perceived as enabling and motivating. Furthermore, having a therapist who was aware and knowledgeable of their personal medical issues or injuries was perceived as important, as the exercises could specifically be tailored to meet their needs.

“I had someone that was a professional that knew about the fact that we all had surgery and so the types of exercises that were given to us was very specific and really tailored towards our own special needs at that particular time.” (P4, Female, 42)

Participants valued having a therapist who was attentive, able to communicate and give feedback whenever needed. This has helped them boost their level of confidence and motivation in the classes. Conversely, failure to address individualized needs proved to be off putting for participants. For example, one participant explained:

“I think the therapist should know each individual capability and what they are fit to do, so that you don't push anyone to a level where they can't do it.” (P2, Male, 58)

The class size was judged to be acceptable, as many participants felt that in a small class, the therapist would be able to better observe and provide personalized guidance and feedback. Participants believed that having too many attendees in each session would have negatively impacted the level of individual attention from the therapist, which may cause participants to be less inclined to attend.

### 3.4 Theme 4: Tele‐exercise provided physical, emotional and social benefits

Overall, all participants reported to have experienced benefits from participating in the tele‐exercise classes including physical, emotional and social benefits. In turn, these perceived benefits appeared to facilitate adherence to the classes and encouraged participants to continue engaging in physical activity beyond the research study. Among physical health benefits reported by the majority of participants are improved fitness, muscle strength, balance and weight loss. Furthermore, most participants believed that the tele‐exercise classes had enhanced their overall psychological wellbeing. They enjoyed the good feeling they experienced after the classes. In particular, the classes helped to take their mind off of what was going on in their lives for that hour of the exercise.

A large number of participants stated that they felt supported, encouraged and motivated by being part of the group of people who had also had bariatric surgery around the same time as them. It opened up the opportunity to share and learn the experiences of others.

“Being able to interact with other people that are using the session, that was good because you could discuss each other journey so far and what they were struggling with or what they found it useful. And then a lot of them would actually share tips with each other on how they do exercise and overcome their challenges.” (P4, Female, 42)

Similarly, a good connection with the exercise therapist also appeared to motivate participants to continue with the classes. This camaraderie between participants and exercise therapist also appeared to facilitate their engagement in everyday physical activity by keeping everyone invested in a shared commitment towards reaching the same exercise goals. However, a small number of participants felt that the tele‐exercise classes had less interaction in comparison to the in‐person classes. In the tele‐exercise classes, they only had the opportunity for brief interaction before and after the classes. Whereas in the gym classes, socializing in person with their exercise peers was perceived as more enjoyable.

Many participants claimed that participation in the tele‐exercise classes had increased their confidence to exercise on their own. For example, some participants bought exercise equipment to add variability to their home exercise routines such as a trampoline, stepper, weights and exercise ball. They also incorporated the exercises learned from the classes into their regular exercise routines. In addition, two participants stated that the programme had prompted them to sign up to online exercise classes after the end of the research programme.

“I've now got online personal trainer who exercise with me. So, all the exercises that I started off with the therapist from our earlier tele‐exercise sessions, I've now developing them even more and more with my personal trainer.” (P12, Female, 55)

## 4 DISCUSSION

This is the first qualitative study to report patients' views and experiences of a home‐based tele‐exercise programme following bariatric surgery. Participants described how tele‐exercise classes helped them to cope with the changes to their lives due to the COVID‐19 pandemic including how it helped them in adhering to the lifestyle change required post‐surgery. Participants found the tele‐exercise schedule, content and intensity to be acceptable, and were satisfied with the privacy, security and safety of the technology and classes. Professional supervision and guidance from the exercise therapist were described as central to the tele‐exercise provision. Importantly, participation in the tele‐exercise provided physical, emotional and social benefits.

A recent survey of 800 patients pre‐ and post‐bariatric surgery supports the current findings of the impact of COVID‐19 on health behaviours and mental wellbeing, as 75% experienced an increased level of anxiety, 60% had decreased levels of physical activity and 30% experienced weight gain.^25^ In fact, during the earlier period of the COVID‐19 outbreak, 7 weeks of lockdown led to 3.8 kg of weight gain with significantly lower weight gain observed in patients who reported performing regular exercise than those who were inactive, 1.1 kg versus 4.6 kg weight gain, respectively.^26^ These observations during the pandemic came to no surprise as physical inactivity and increased sedentary behaviour following bariatric surgery are associated with poor long‐term weight loss outcomes.^27^

Overall, participants in the present study reported positive views and experiences of the tele‐exercise classes. The structure, content and intensity of the tele‐exercise classes were perceived as equally acceptable and satisfactory as the in‐person gym classes they attended prior to COVID‐19 pandemic. Enjoyment and a positive experience of exercise have been shown to increase exercise adherence and motivation,^11^, ^28^ which in the case of the present study, appeared to contribute to the changing perceptions of home‐based exercise. In addition, some participants expressed better confidence level in the tele‐exercise classes, compared to how they previously felt during in‐person gym classes. It has been previously reported that self‐consciousness about physical appearance when exercising in public spaces and exercise facilities did not disappear despite significant weight loss after bariatric surgery,^24^ therefore tele‐exercise classes may enable patients to overcome this barrier to exercise post‐surgery.

The increased accessibility of tele‐exercise compared to in‐person classes overcome several barriers faced by patients to engage in physical activity such as lack of time, geographical accessibility of exercise facilities and poor weather, which is in line with findings from previous studies.^23^, ^24^, ^29^ An earlier feasibility study that evaluated an in‐home supervised exercise programme via telehealth in patients awaiting bariatric surgery reported greater attendance compared to the in‐hospital supervised exercise programme (95.8% versus 80.1%, respectively).^30^ In the present study, the problem associated with the access to the tele‐exercise classes and technical setup of software were minimal. This is unsurprising as of 2020, 96% of households in the United Kingdom having internet access.^31^ Additionally, 83% of participants in this study were from a higher educational background, with an age below 65 that was deemed to be technology savvy.^32^

People living with obesity generally experience a wide range of barriers to engaging in physical activity encompassing both internal barriers, which can be divided into physical (excess weight, poor fitness, health problems, injury) and psychological barriers (weight perception, low mood, lack of enjoyment and motivation/willpower), and also external barriers (lack of time and knowledge, poor weather, competing demands).^33^ Despite a significant weight loss following bariatric surgery, the majority of these barriers to exercise continue to persist.^24^ Ongoing support from an exercise professional is therefore recommended particularly following bariatric surgery^29^ and our data suggest that this was needed during the COVID‐19 pandemic, which created further barriers to engaging in physical activity. In the present study, participants emphasized the important role of the exercise therapist both in providing an exercise programme tailored to their physical capacity and in supporting them to tackle psychological barriers. A study by Bergh et al. has recently highlighted the importance of interventions targeting patients' abilities to make plans, enhance self‐efficacy and improve action control skills as they found a strong relationship between these self‐regulation factors with both objective and self‐reported physical activity after bariatric surgery.^34^

To date, a growing number of studies have attempted to elucidate the beneficial effects of exercise programmes following bariatric surgery in order to support a post‐surgery exercise recommendation.^5^ Although the effect of exercise post‐bariatric specifically in enhancing weight loss remains inconclusive due to the paucity of high‐quality studies,^35^ several other positive outcomes were reported hence favoured recommendation, including preventing excessive loss of fat‐free mass, enhancing physical and cardiorespiratory fitness, promoting better health‐related quality of life, among other benefits.^36^, ^37^ In the present study, participants perceived that the tele‐exercise not only benefited them in terms of physical health but also their social and emotional wellbeing were improved. Notwithstanding, high‐quality studies of tele‐exercise following bariatric surgery that measure these reported outcomes using objective assessment tools are still needed to confirm these early findings.

### 4.1 Practical considerations for future implementation

To optimize the delivery of tele‐exercise, several important aspects should be taken into consideration. Using a hospital‐based virtual platform to deliver the tele‐exercise would be a better option as this will assure participants of their privacy and safety being well‐protected. Providing participants with clear written guidance such as login instruction will ensure a smooth process of the tele‐exercise delivery. For a clear visual and access, ideally, participants would require a device with a larger screen (e.g., desktop, laptop or tablet) and a minimum internet bandwidth required to access a virtual platform. To ensure tailored and personalized supervision, the class size should be limited ideally between five to eight participants per session. Furthermore, an initial in‐person session with an exercise therapist prior to enrolment in tele‐exercise is needed to assess participants' exercise capacity for tailored exercise prescription and building rapport. Recording the tele‐exercise classes with availability to access this resource on demands were found to be useful but the copyright of the recordings should be taken into consideration. As per participants' suggestions, consider providing other simple and cheap exercise tools such as a yoga mat and exercise ball which were thought to be suitable and applicable for the tele‐exercise. Finally, regarding the scheduling, a mix of weekdays and weekend options covering morning and evening classes would increase the likelihood of attendance. For the evening class, consider a later evening time to provide an opportunity for patients who are working in a daytime to get ready for the tele‐exercise.

### 4.2 Strengths and limitations

The present study captured in‐depth views and experiences of patients towards a tele‐exercise intervention following bariatric surgery and included a varied sample of male and female participants, with a wide age range and diverse ethnic backgrounds. We assumed that a sample of 12 participants was deemed appropriate because of the early, exploratory nature of this study and the focus was to gain preliminary insights that were useful for the planning and development of future robust tele‐exercise interventions. However, as the majority of participants in this study were highly educated and employed, the generalisability is somehow limited. Digital exclusion, especially among patients from a lower socio‐economic group, may impact the uptake of such programmes.^38^ In the current climate of the COVID‐19 pandemic and associated restrictions, tele‐exercise might be perceived positively, and as beneficial. Therefore, patients' perceptions towards tele‐exercise delivered during a non‐pandemic period should be further explored. In addition, the present study did not explore the views and experiences of the exercise therapists, which are important to consider when designing and implementing future tele‐exercise interventions. Lastly, although the majority of participants perceived the tele‐exercise to be as effective as the in‐person classes, we recognize that a quantitative study that objectively compares both methods is required in order to support the present findings.

## 5 CONCLUSIONS

The COVID‐19 pandemic has revolutionized the way healthcare is provided through telehealth. The present study suggests that tele‐exercise, when implemented specifically in patients who have undergone bariatric surgery, is feasible and well‐accepted, and potentially as effective and useful as in‐person exercise classes. These preliminary findings have provided additional insights into much‐needed evidence for the potential use of telehealth in the provision of care following bariatric surgery.^17^ In today's technologically advanced society, it is foreseeable that telehealth will eventually become a new norm for future healthcare. Therefore, it is timely and relevant now to undertake more robust research designs to investigate the efficacy and effectiveness of tele‐exercise pre‐ and post‐bariatric surgery. The research findings will be not only useful to face the present and future pandemics but can also be translated and integrated into the existing bariatric care pathway to optimize patient outcomes.

## AUTHORS CONTRIBUTIONS

**Friedrich C. Jassil**: conceptualisation; data curation; participant recruitment; formal analysis; writing. **Rebecca Richards**: formal analysis; writing. **Alisia Carnemolla**: conceptualisation; writing. **Neville Lewis**, **Helen Kingett**, **Jacqueline Doyle**, **Amy Kirk**, **Adrian Brown**: intervention. **Gemma Montagut‐Pino** and **Kusuma Chaiyasoot**: data curation. **Kalpana Devalia** and **Chetan Parmar**: principal investigator. **Rachel L. Batterham**: chief investigator; conceptualisation; data curation; funding acquisition; resources; writing. All authors read and approved the final manuscript.

## Supporting information


**Appendix** **S1**. Supporting Information.
