# Supplementary material for: Patients' views and experiences of live supervised tele‐exercise classes following bariatric surgery during the COVID‐19 pandemic: The BARI‐LIFESTYLE qualitative study
Source: Clin Obes. 2021 Nov 28;12(2):e12499. doi: 10.1111/cob.12499 (PMC9011650; doi:10.1111/cob.12499)
Supplement: Supplementary file 1 — Appendix S1. Supporting Information. [file COB-12-e12499-s001.docx]

**Supplementary 1:** Tele-exercise protocol

Each tele-exercise class lasted for 60 minutes, consisted of 10 minutes warm-up, 40 minutes of a combined aerobic and resistance training, and 10 minutes of cool down. In all classes, the exercise therapists performed the exercises and interacted with participants in real-time. The warmup and cool down period involved gentle mobilising and muscle stretching, mimicking some yoga- and Pilates-style movements. The main exercise session consisted of aerobic training which involved a variety of exercises aimed to increase heart rate that was mixed up with resistance training targeting all major muscles. The three types of resistance bands (PhysioRoom.com, UK) used during the classes were provided to participants on their first in-person exercise class pre-lockdown. The type of exercises using the resistance bands included exercises such as biceps curls, tricep extensions, overhead press, front and lateral raises, and reverse flies. The exercise therapist also used body weight to perform the resistance exercises such as squats, lunges, press ups and planks. The resistance exercises were performed in three sets with between eight to ten repetitions each. Exercise intensity was adjusted by increasing the pace and duration of the exercises, based on individual fitness and functional capacity as deemed necessary by the exercise therapist.

**Supplementary 2:** Semi-structured interview guide

| **Main question** | **Probing questions** |
| --- | --- |
| Can you share your experience on the tele-exercise that you took part during the COVID-19 pandemic? | - Were there any benefits of the tele-exercise? - Did it have impacts on your physical and mental wellbeing? - How was the scheduling and the length of each class? - Did the classes help you cope with the pandemic? |
| What do you think about the use of telecommunication technology to deliver the exercise programme? | - Can you comment on the installation process, the device you used and your internet connection? - Did you experience any technical issues or any other difficulties using this platform? - Did you have any privacy concerns using this platform? - What do you think about the video recording of the classes? - Can you comment on the number of participants in each class? |
| Can you share your view about the content of the tele-exercise classes? Did the classes meet your expectation? | - How was the intensity compared to the gym classes that you have attended? - Was there any aspect of exercise you liked most/least enjoy and easiest/hardest to do? What were they and why? - Do you have any suggestions on the type of exercise we should do more in the class? - How confident were you to perform the exercise remotely? Did you have any concern of getting yourself injured? - Do you think the resistance band we gave enough? Any other home-based exercise equipment you would like to suggest giving to participants? - Can you comment on the space at home to do the exercise? - Did you do any extra exercise on your own based on what you have learned in the classes? |
| Can you share about the level of supervision you received from the therapist in the classes? | - Did you have the opportunity to give feedback about what you feel about each class? - If you have any concern for example injury, do you think that was being addressed? - Was it easy to communicate with the therapist throughout the tele-exercise classes? |
| Can you share if there are any enablers or any barriers that you faced in participating in the weekly class? | - Any support you received from family members, the tele-exercise group or therapist that facilitated your attendance? - Any strategies or personal skills that you found helpful that enabled you to attend the classes? - For the classes that you were unable to attend, what were the reasons? |
| In the future, does tele-exercise could be an effective way to deliver the programme? | - From your experience in attending both the gym and tele-exercise classes, can you share the pros and cons of both programmes? - Any aspects of the exercise you continue doing beyond the study? Which one and to what extent? - What do you think of the tele-exercise to be included as part of standard care after bariatric surgery? - Any suggestions to improve the tele-exercise provision in future? |

**Supplementary 3**: Examples of quotations for themes and subthemes

| **Themes** | **Sub-themes** | **Examples** |
| --- | --- | --- |
| Coping with the impact of COVID-19 lockdown | Anxiety around maintaining weight-management behaviours | *“Especially now we've got this second lockdown, finding that I'm going to struggle to find times to exercise or be motivated to exercise.” (P4, Female, 42).* |
|  | Exercise alone at home was anticipated to be difficult | *“For me, because of COVID and everything, I'll be exercising by myself at home. I don't like to exercise alone. I don't like it at all.” (P10, Female, 35)* |
|  | Limited social interaction impacted mental health | *“Especially at that point in the lockdown, you were told to go outside only if it’s essentials. So, it [the tele-exercise] was an outlet, it stabilised mood, it kept you active. It provided a lot of help.” (P7, Male, 33)* |
|  | Classes facilitated the self-regulation of exercise | *“It was good to have a bit of a structure during the lockdown that having sort of diary appointments. It encouraged me to do more exercise and push myself harder during lockdown and have structure.” (P9, Female, 37)*  *“Sometimes when I didn't really feel like it, then I think sort of knowing that there was a small group who would be aware that I wasn't there, would encourage me to log on.” (P9, Female, 37)* |
| Tele-exercise programme was perceived as acceptable | Challenges and solutions associated with the technology | *“We didn't have any issues getting linked to the Zoom. I think everyone is doing it now. I think it's quite easy and quite commonly used.” (P2, Male, 58)*  *“It was a little confusing about the passwords at first, but I mean it wasn't terribly complicated once you understand how to use Zoom.” (P5, Male, 63)*  *“It was hard for me because I suppose to watch the class from phone. But then I moved on to the laptop and much better.” (P1, Female, 39)*  *“I only have problems one time and it was when I was trying to do it outside the house, so the signal wasn't so good, but I wanted the bigger space to move.” (P3, Male, 52)*  *“In the early days, my screen used to freeze, and I'd be thrown out and had to log back in. Once I changed over the amount of Internet that got into the house it was much better.” (P12, Female, 55)*  *“Maybe send them an email about the ‘how-to’ guide just to make sure everyone is up to speed. Because that way, you can get straight into the session if there were no technical issues.” (P7, Male, 33)* |
|  | Satisfaction towards the tele-exercise structure, content and intensity | *“All the exercises were quite good. [The therapist] worked every kind of muscle, with the legs, hands, neck, back, shoulders. [The therapist] was multi choices as well, using bands, carpet [floor exercises], all the heavy things, and doing a lot of stretching. I think it was kind of including Pilates and yoga.” (P1, Female, 39)*  *“It was absolutely just right because there's only so much exercise that you could fit in that you can get everyone's participation. I’d say if you did it any longer, I think you'd lose people.” (P8, Female, 55)*  *“Even though I wanted more but 60 minutes I think is nice for everyone. If I would do it again, I will ask for more time because after 60 minutes, I have more energy.” (P10, Female, 35)*  *“I think the 60 minutes was fine. You got enough wear out and was ready to rest after the 60 minutes.” (P6, Female, 48)*  *“Especially like people who maybe haven't used the gym much or not for many years, I think it's an important aspect to do this sort of face-to-face and trying to get people in the gym and develop those healthy habits and give people confidence in the gym.” (P9, Female, 37)*  *“Ideally, a combination of the two [in-person and tele-exercise] would be the perfect thing.* *Perhaps the first session could be at least in person, to let the therapist understand your level of exercise, your ability to exercise. And then, if you're not using weights, I just don't even see why you have to be in person. You can probably reach so many more people.” (P3, Male, 52)* |
|  | Positive changes on the perceptions of home exercise | *“At first I was a little bit apprehensive because I've never done anything like that before. But after doing the first, I couldn't move. So, for the following sessions what I found was, yeah, I'm enjoying this.” (P8, Female, 55)*  *“Being able to get such a good full workout in the house changed my whole perspective on what was possible from home. [The therapist] showed me you can work out in kind of ‘a small little box’.” (P3, Male, 52)*  *“Something about the gym is you don't learn a lot of the different kinds of ways to exercise various muscles because you do over-rely on the machine. So that's why I said about learning new exercise regimes from the Zoom sessions. It allows you to learn what you would do at the gym, how to do at home in a much simpler way.” (P7, Male, 33)* |
|  | Tele-exercise was viewed as private, secure and safe | *“I think it [tele-exercise] is definitely the way forward, especially as an individual who may feel self-conscious about yourself in the way that you look when you exercise. In that sense, it doesn't debilitate your confidence rather than a booster. It’s brilliant”. (P12, Female, 55)*  *“I think, for what we were doing, the level of privacy is fine, and it included a password. I know there are some larger issues around Zoom, but I don't feel that we were doing something particularly private.” (P11, Female, 39)*  *“I think it would be better off if the hospitals or the NHS device something that they can use solely for patients, then that would be a good idea.” (P8, Female, 55)*  *“I was a little bit concerned with some of the exercises, but I think that the difference here is that I'm not forced to do them. So, where I found I knew already that carrying out those core exercises, I would probably cause myself more injury or damage because of the medical conditions I previously had, then I would leave them out.” (P4, Female, 42)* |
|  | Tele-exercise removed some barriers to accessing structured exercise classes | *“It [tele-exercise] made exercise very accessible to me. Of course, one of the big advantages of it is that I could take it abroad. I did the exercise class in [country name] and that was great.” (P5, Male, 63)*  *“I think, maybe for the evening, to move it till 6 pm, but I don't know if that works for everybody.” (P9, Female, 37)*  *“I'm a key worker, and during this lockdown, I went to work. And sometimes I was supposed to do overtime and stuff, and that was the reason I couldn't attend to all of it” (P1, Female, 39)*  *“My own health was the biggest barrier and my stress level. They’re particularly biggest barriers for me.” (P11, Female, 39)*  *“I would certainly make recordings available and find a clear way of formalising it. I'm aware that there are maybe copyright issues that need to be sorted, but I'm sure it's possible to sort that one out.” (P5, Male, 63)* |
| Professional supervision and guidance affecting adherence to tele-exercise |  | *“You can tell that while exercising you were being watched and if you found it difficult, the therapist would say ‘are you okay? if you can't do it this way, you can do it this way’. It was very good communication as well.” (P3, Male, 52)*  *“[The therapist] was a good listener. Whenever I asked something, [the therapist] will answer me directly and will give me options if the exercise was difficult to do. [The therapist] is amazing.” (P10, Female, 35)*  *“I think a session will probably a maximum of eight. Anything bigger than that, I think I would have probably felt like less involved or less likely it was actually tailored towards my needs.” (P4, Female, 42)*  *“I think if it was a lot much larger group then people might be less inclined to attend, but as it was a small group then it was very positive.” (P9, Female, 37)*  *“If you have too many, it becomes less personal and if you have too few, it can feel a bit off. Too many people, the therapist would have trouble spotting things like people’s technique.” (P7, Male, 33)* |
| Tele-exercise provided physical, emotional and social benefits |  | *“I did it once a week, but it has an amazing impact. My body has changed especially I'm losing a lot of weight very quick. That helped a lot.” (P10, Female, 35)*  *“Obviously it [the tele-exercise] helped with weight and build up muscle mass, and that because when you've had surgery you lose a lot of muscle mass.” (P6, Female, 48)*  *“It helped me keep fit and it took your mind off of what was going on for that hour of the exercise. You were due for an hour of what was going on around you.” (P6, Female, 48)*  *“Having that interaction with the therapist and meeting with the group on a weekly basis, you have something to look forward to. I found it was emotionally empowering as well as mentally.” (P4, Female, 42)*  *“It's very good and it's nice because you could see the other people, [the therapist], and they could see me too. You could have a chat with them so you can sort of connect and support each other.” (P11, Female, 39)*  *“But I prefer the session in the hospital because I prefer socialising with others in person than via online, it's convenient to be in the gym.” (P1, Female, 39)*  *“Now I have a personal trampoline, I have a personal stepper, I have weights, all these things so I can exercise in the house. It really changed my idea of what was possible.” (P3, Male, 52)*  *“The tele-exercise has increased my confidence to exercise by myself. I've incorporated press-ups, lunges, squats, and things into my running. It's not just about confidence, it's more about motivation and keeps going.” (P9, Female, 37)* |
